# The Role of Endothelial Progenitors in the Repair of Vascular Damage in Systemic Sclerosis

**DOI:** 10.3389/fimmu.2018.01383

**Published:** 2018-06-18

**Authors:** Nicoletta Del Papa, Francesca Pignataro

**Affiliations:** Department of Rheumatology, ASST G. Pini-CTO, Milano, Italy

**Keywords:** systemic sclerosis, endothelial progenitors, stem cells, vasculogenesis, angiogenesis

## Abstract

Systemic sclerosis (SSc) is a connective tissue disease characterized by a complex pathological process where the main scenario is represented by progressive loss of microvascular bed, with the consequent progressive fibrotic changes in involved organ and tissues. Although most aspects of vascular injury in scleroderma are poorly understood, recent data suggest that the scleroderma impairment of neovascularization could be related to both angiogenesis and vasculogenesis failure. Particularly, compensatory angiogenesis does not occur normally in spite of an important increase in many angiogenic factors either in SSc skin or serum. Besides insufficient angiogenesis, the contribution of defective vasculogenesis to SSc vasculopathy has been extensively studied. Over the last decades, our understanding of the processes responsible for the formation of new vessels after tissue ischemia has increased. In the past, adult neovascularization was thought to depend mainly on angiogenesis (a process by which new vessels are formed by the proliferation and migration of mature endothelial cells). More recently, increased evidence suggests that stem cells mobilize from the bone marrow into the peripheral blood (PB), differentiate in circulating endothelial progenitors (EPCs), and home to site of ischemia to contribute to *de novo* vessel formation. Significant advances have been made in understanding the biology of EPCs, and molecular mechanisms regulating EPC function. Autologous EPCs now are becoming a novel treatment option for therapeutic vascularization and vascular repair, mainly in ischemic diseases. However, different diseases, such as cardiovascular diseases, diabetes, and peripheral artery ischemia are related to EPC dysfunction. Several studies have shown that EPCs can be detected in the PB of patients with SSc and are impaired in their function. Based on an online literature search (PubMed, EMBASE, and Web of Science, last updated December 2017) using keywords related to “endothelial progenitor cells” and “Systemic Sclerosis,” “scleroderma vasculopathy,” “angiogenesis,” “vasculogenesis,” this review gives an overview on the large body of data of current research in this issue, including controversies over the identity and functions of EPCs, their meaning as biomarker of SSc microangiopathy and their clinical potency.

## Introduction

Systemic sclerosis (SSc) is a chronic connective tissue disease of unknown etiology characterized by immunologic abnormalities, microangiopathy, and excessive deposition of collagen in the skin and different internal organs (1). Clinical and pathologic findings of vascular damage and endothelial activation strongly support the hypothesis that the vascular involvement could be the most important and the primary process in the pathogenesis of scleroderma (2). Morphological changes in the vessels of patients with SSc range from considerable initial derangement with capillary thrombosis, to often abortive reparative neoangiogenesis with abnormal capillary proliferation, and to almost complete loss of vessels with persistent ischemic injury in target tissues (3, 4). Although tissue hypoxia is a strong inducer of neovascularization, new microvessel formation appears to be defective in SSc patients and no evidence exists of an effective replacement of damaged capillaries (5). Recent data suggest that the scleroderma impairment of neovascularization could be related to both angiogenesis and vasculogenesis failure (6). Angiogenesis is defined as the creation of new vessels sprouting out of pre-existing ones, whereas vasculogenesis refers to the *in situ* formation of blood vessels from hemangioblasts or vascular stem/progenitor cells (7, 8). In SSc, serum levels of both pro-angiogenic mediators and powerful inhibitors of angiogenesis are largely alterated, especially in the active phases of the disease. In addition, abnormalities in pro-angiogenic signal transduction pathways have been reported, suggesting an intrinsic impaired response of SSc endothelial cells to the mechanisms of vascular angiogenic repair (9). In this scenario, the endothelial cell apoptosis could be recognized as an additional feature of disturbed angiogenesis (10, 11).

In contrast to angiogenesis, during vasculogenesis the formation of new blood vessels can occur in the absence of pre-existing blood vessels through the recruitment and differentiation of endothelial progenitor cells (EPCs). Interest in EPC biology has been growing continuously since their discovery (12) and now EPCs are regarded as biomarkers in cardiovascular diseases and also potential sources of cell for revascularization strategies, which may include direct cellular transplantation and tissue engineering. Significant advances have been made in understanding the biology of EPCs, and preclinical studies using transplanted EPCs provided promising results in the treatment of ischemic diseases (13). Altogether, in the last decade, these data have given rise to several studies regarding the role of EPCs in SSc vasculopathy. The purpose of this review is to evaluate the relevant scientific literature to determine the current state of knowledge on EPCs in the context of the scleroderma vasculopathy. We conducted an online literature search (PubMed, EMBASE, and Web of Science, last updated December 2017) using keywords related to “endothelial progenitor cells” and “Systemic Sclerosis,” “scleroderma vasculopathy,” “angiogenesis,” “vasculogenesis.” Eligible papers were evaluated on four pertinent criteria: (1) SSc study populations and appropriate controls, (2) markers used to define EPC phenotype, (3) methods used for assessing EPC function, and (4) evaluation of the possible correlation between EPC detection and angiogenesis/vasculogenesis processes in SSc.

Herein, we summarize the pertinent findings of these studies and discuss the potential role of EPCs as biomarker of scleroderma microangiopathy, the controversies over the identity and functions of EPCs, and their potential clinical potency.

## Characterization and Biology of EPCs

The term “Endothelial Progenitor Cells” (EPCs) should be basically used to refer to populations of cells that are capable of differentiation into mature endothelial cells in vasculogenesis (*de novo* formation of vascular networks) (14). Many studies have attempted to identify cell surface markers that are unique to EPCs and distinguish them from mature endothelial cells. EPCs were identified for the first time in 1997 by Asahara et al. in human peripheral blood (PB) as a subset of hematopoietic cells with vasculogenic properties *in vivo* and *in vitro* (12). They identified these cells as CD34^+^ (a protein with unknown function expressed in early hematopoietic cells) and KDR^+^ [kinase-insert domain containing receptor that encodes for vascular endothelial growth factor receptor 2 (VEGFR2)]. Since CD34^+^/VEGFR^+^ cells may also identify circulating mature endothelial cells shed from damaged vessel, subsequent works have included CD133 as the stemness marker of EPCs (15). However, the use of CD133 remains controversial. Case et al. showed that mobilized adult PB CD34^+^/VEGFR2^+^/CD133^+^ cells represent an enriched population of CD45^+^ hematopoietic precursors, which do not differentiate into endothelial cells *in vitro* (16). Other authors suggested VE-cadherin and E-selectin as additional surface markers to identify progenitor cells in a more advanced stage of endothelial maturation (17). Due to this controversial scenario, it is evident that the family of EPCs is characterized by lineage and functional heterogeneities, with a spectrum of phenotypes not yet fully defined.

Another approach to EPC isolation and characterization is represented by the use of defined culturing assays to culture unselected PB mononuclears cells (MNCs). For EPCs isolated by cell culture assays, there is now agreement that two different populations can be identified. Hur et al. (18) plated MNCs on a fibronectin- and/or gelatin-coated plate with VEGF containing medium to obtain two types of EPCs, early and late EPCs. Early EPCs with spindle-shaped morphology showed peak growth at 2–3 weeks, whereas late EPCs, cobblestone-shaped, showed exponential growth at 4–8 weeks. The definition of early and late EPCs, based on their time of appearance in culture, reflects a very different phenotype, one being hematopoietic and the other endothelial, respectively (19). Recently, Medina et al. recommended identifying in early EPCs myeloid angiogenic cells (MACs) to clarify their lineage and function. MACs are defined as cultured cells derived from PB mononuclear cells which are grown under endothelial cell culture conditions. These cells share multiple surface antigens of monocytes (CD45, CD14, CD31) and are negative for CD133, CD146, and Tie2 (20). Most studies suggest that these short-term cultures of MNCs fail to differentiate themselves into functional endothelial cells, but predominantly promote vessel formation by activating resident endothelial cells through paracrine mechanisms (21–23).

Late EPCs are often called out growth EPCs and have a more mature phenotype. These cells lack hematopoietic and myeloid markers and are usually derived from long-term cultures of at least 2–4 weeks *in vitro* (24, 25). They are now commonly named as endothelial colony-forming cells. They generate mature endothelial progeny *in vitro* and it has also been observed that they physically contribute to formation of new capillaries (26, 27).

The exact definition and characterization of EPCs is still an on-going and unresolved issue. EPC populations represent a heterogeneous mix of progenitors, in terms of lineage, proliferative potential, and mechanism of action. Experimental models suggest that there is no evidence for the superiority of one cell type over another, and different signaling pathways co-work in regulating EPC commitment (28). Furthermore, Yoon et al. showed that *in vitro*, the angiogenic capability of the two cell types (early and late EPCs) was augmented by mutual interaction through cytokines and metalloproteinases. In addition, the injection of a mixture of the two cells resulted in a superior neovascularization than that obtained by using any one of the single-cell-types (29).

The characteristics of the *in vivo* environment may influence the fate and function of EPCs. In normal homeostatic conditions, there is a low number of circulating EPC populations in the PB. EPCs reside within a stem cell niche in the bone marrow (BM) and complex mechanisms regulate EPC trafficking from the BM to the bloodstream (30). In reality, mechanisms that trigger the regeneration process of EPCs are not well understood and are still under investigation. Tissue ischemia is believed to be the most powerful physiological stimulus for mobilizing EPCs from the BM to the site of new vessel growth (31, 32). EPC recruitment requires a coordinated sequence of multi-step adhesive and signaling events, including chemoattraction, adhesion, and migration. Once at the site of tissue repair, EPCs contribute to new vessel formation in different ways: direct incorporation into the neovessel wall, differentiation into mature endothelial cells, and production of paracrine signals including growth factors, such as VEGF, stromal derived factor, monocyte chemotactic protein 1, and platelet-derived growth factor. Altogether, these pro-angiogenic molecules might further activate resident endothelial cells toward proliferation and vascular repair (28, 33).

## EPCs in SSc

In spite of the on-going controversy over EPC identity, the clinical significance and therapeutic potential of EPCs in vascular regenerative applications has been extensively studied in the last few decades. The existence of postnatal vasculogenesis, mediated by a population of endothelial progenitors identified in both the BM and PB, represents an important tool to better understand the biological contribution of EPCs in different vascular diseases, including SSc.

Several studies have demonstrated that the number and function of EPCs (both in circulation and BM) may be impaired in some disorders characterized by prolonged chronic endothelial damage, such as chronic coronary artery disease, hypertension, congestive heart disease, and diabetes mellitus (34, 35). It has, therefore, been postulated that impaired vascular repair may contribute to the pathogenesis of these chronic disorders and that a small number of EPCs may also be a risk factor for atherosclerotic plaque instability (36). By contrast, an increased number of EPCs is often observed in patients experiencing a myocardial infarction (37) or acute vascular trauma (38). Several studies have suggested that EPCs have a role in the pathogenesis of different autoimmune diseases (34, 39). On average in rheumatoid arthritis (RA) and systemic lupus erythematosus (SLE), studies have found decreased peripheral counts of EPCs with altered function (39). The interpretation of these observations is quite difficult at the moment because of the presence of an intrinsic increased cardiovascular risk related to these inflammatory disorders that alone might be responsible for EPC involvement.

Several studies have shown that EPCs can be detected in the PB of patients with SSc and are impaired in their function (40–44). However, the numbers and functions of EPCs in SSc is still a matter of debate, since conflicting reports have been released. Whereas some studies reported a significant depletion in the count of CD34^+^/CD133^+^/VEGFR2^+^ cells in SSc patients using flow-cytometry (40, 45–47), other studies found increased circulating EPC counts, mainly in the early and active stages of the disease (41–43). By contrast, lower EPC counts were associated with long disease duration, higher Medsger’s severity scores, and ischemic digital ulcers (41–44). These different findings may be ascribed to difficulties in correctly assessing patients with different scleroderma subtypes, disease duration, and level of disease activity.

The complexity of this scenario is further increased by the identification of a subpopulation of multipotent circulating monocytes able to differentiate along the endothelial lineage and promote neovascularization *in vitro* and *in vivo* (48). Really, the current consensus is that these monocytic cells, termed monocytic pro-angiogenic hematopoietic cells (PHCs) and characterized by the positive expression of CD14, CD45, CD34 and type I collagen, do not give rise to endothelial cells *in vivo*, but can support vascular repair through their rapid recruitment to the site of endothelial injury, local secretion of pro-angiogenic factors, and differentiation into mural cells (49). Increased circulating PHCs have been found in patients with SSc (50, 51) and some correlation with the fibrotic clinical features of the disease was observed (50).

Recent studies showed that a specific T cell population, called angiogenic T cells (Tang) may contribute to the formation of new vessels enhancing endothelial cell proliferation and function. Hur et al. demonstrated that these T cell subtype, characterized by the surface phenotype CD3^+^CD31^+^CXCR4^+^, constituted the center of EPC colonies during cultures of human PB mononuclear cells. These Tang were required for colony formation and differentiation of early EPCs, actively participating in postnatal vasculogenesis and vascular repair (52). Clinical studies showed an inverse relationship of Tang with cardiovascular risk factors, even in autoimmune diseases, such as RA, SLE, and ANCA-associated vasculitis (53–55). In SSc, Tang cell counts have been reported to be selectively increased in the PB of SSc patients with severe vascular complications like digital ulcers and there was an inverse correlation between circulating Tang and EPCs (defined as CD34^+^CD133^+^VEGFR2^+^). Furthermore, the presence of circulating Tang cells positively correlated with the levels of pro-angiogenic factors, namely VEGF and MMP-9 (56). Such Tang cell expansion has, therefore, been suggested as a possible ineffective attempt to compensate the decrease in EPC number (56).

Indeed, a growing body of evidence suggests that there is a large heterogeneity of EPC populations, which include both hematopoietic and non-hematopoietic lineages. Thus, different subtypes of circulating progenitor cells with vasculogenic and/or angiogenic potentialities have been identified in patients with SSc (see Table 1). However, the methods of characterization and quantification of EPCs are not standardized, and the protocols used to count EPCs vary in the different studies (57). Methodological issues are further increased by the difficulties in reliably identifying EPCs. In view of this complexity, the European League against Rheumatism Scleroderma Trials and Research (EUSTAR) recommendations appear to have a limited application (58).

**Table 1:** Circulating EPCs detected in systemic sclerosis (SSc).

Surface markers detected by FACS analysis	Alterations in EPC number	Association with SSc clinical features	Reference
CD34^+^/CD133^+^/VEGFR2^+^	Decreased	Active digital ulcers and pitting scars	Kuwana et al. (40)

CD34^+^/CD133^+^	Increased in the early phase, decreased in the late phase	None	Del Papa et al. (41)

CD34^+^/CD133^+^/VEGFR2^+^	Increased in the early phase, decreased in the late phase	None	Del Papa et al. (59)

CD34^+^/CD133^+^	Increased	European disease activity score	Allanore et al. (42)

CD34^+^/CD133^+^; CD34^+^/CD133^+^/VEGFR2^+^ CD34^+^/VEGFR2^+^	Decreased	None	Zhu et al. (45)

CD34^+^/VEGFR2^+^ CD133^+^/VEGFR2^+^	Increased in the early phase, decreased in the late phase	Severity of peripheral vascular manifestations in the early, severe organ involvement in the late	Nevskaya et al. (44)

CD34^+^/VEGFR2^+^ CD133^+^/VEGFR2^+^	Decreased	None	Mok et al. (46)

Lin^−^/7AAD/CD34^+^/CD133^+^/VEGFR2^+^	Increased	Inverse correlation with Medsger’s severity score and Digital Ulcers	Avouac et al. (43)

CD34^+^/CD133^+^/VEGFR2^+^ CD34^+^/VEGFR2^+^ CD133^+^/VEGFR2^+^	Decreased	None	Andrigueti et al. (47)

CD133^+^/VEGFR2^+^	Decreased	ND	Patschan et al. (88)

Additional *in vitro* studies showed that, despite having a normal phenotype, EPCs isolated from SSc patients had an impaired ability to differentiate into mature endothelial cells (40). These functional changes have been further confirmed by the examination of BM aspirated from SSc patients that showed low numbers of EPCs characterized by impaired capacity for endothelial differentiation (59). Interestingly, only the BM EPCs from patients with early disease led to some degree of endothelial differentiation, thus suggesting a probable progressive exhaustion of the pool of the BM resident EPCs during the disease progression. The increased levels of VEGF observed in SSc sera (40, 59–62) and the high expression of VEGF receptor on the surface of BM EPCs generated the intriguing hypothesis of a powerful angiogenic “push” without an appropriate vessel formation in SSc patients. Really, no study has shown a direct evidence of correlation between VEGF levels and circulating EPCs in SSc patients. In addition, recent studies have shown that the “VEGF scenario” is rather more complex in SSc and cannot be simply explained by a general insufficient angiogenic/vasculogenic response to important promoters of new vessel formation. Indeed, the overexpression of the anti-angiogenic splice isoform VEGF_165_b in the plasma, microvascular endothelial cells dermis, and in the platelets of SSc, adds a new play actor in the set of the mechanisms of scleroderma dysfunctional angiogenesis (63–65).

However, the hypothesis of a direct link between EPCs and pro-angiogenic soluble factors has been further sustained by recent data showing a positive correlation between EPC mobilization and circulating levels of Fractalkine in a context of endothelial activation in SSc patients (66). Fractalkine, the only member of the CX3C chemokine family, has recently been described as an angiogenic chemokine. Previous studies have shown that fractalkine induced endothelial cell migration and proliferation, EPC migration and tube-like structure formation *in vitro*, and stimulated new blood vessel formation *in vivo* even in the context of ischemic diseases (67, 68).

An alternative to the hypothesis that prolonged and continuous endothelial cell recruitment may exhaust the BM reservoir of resident EPCs is that disease-related toxic mechanisms can negatively influence the half-life and mobilization of BM EPCs. Since EPCs share the phenotypic and functional properties of mature endothelial cells (15), it is possible that the same immuno-mediated mechanisms capable of inducing peripheral endothelial injury in SSc (i.e., apoptotic phenomena and/or anti-endothelial activity) might also be found in the BM environment. This hypothesis has been confirmed by the detection of significant titers of anti-endothelial cell antibodies (AECA) in the SSc BM plasma (69). Furthermore, their presence correlated with that of activated and apoptotic progenitors (59, 69). These findings were further confirmed by an *in vitro* assay in which the apoptosis of normal progenitors was induced by the addition of AECA^+^ purified IgG (69). The role of apoptosis in SSc endothelial impairment, even at progenitor level, has been further elucidated by Zhu et al. (45). They reported an increase rate of apoptosis in EPCs isolated from PB of patients with SSc. Depletion of IgG fraction from SSc sera completely abolished the apoptotic effects, suggesting that EPCs may be destroyed upon their mobilization from the BM by serological factors, may be auto-antibodies, present in the SSc sera. As expected, SSc sera induced apoptosis even in human mature microvascular endothelial cells, although these cells were less susceptible than EPCs to the toxic factors present in the SSc sera (45). Similar to what happens in other cell types, the Akt-FOXO3a–Bim axis is the key pathway implicated in apoptotic process in SSc EPCs (45). In summary, these findings support the hypothesis that AECA may play a pathogenetic role by affecting the BM EPC machinery that should repair the peripheral vascular lesions.

Recently, Shirai et al. proposed Pentraxin 3 (PTX3) might represent another intriguing candidate as a contrasting mediator of EPC-mediated vasculogenesis in SSc (70). The expression of PTX3 is induced by inflammatory cytokines in response to inflammatory stimuli in several mesenchymal and epithelial cell types, particularly endothelial cells and mononuclear phagocytes. Additional properties of PTX3 other than those related to innate immunity and inflammation have been described, such as extracellular matrix deposition (ECM), tissue remodeling, and angiogenesis. PTX3 is involved in a variety of molecular mechanisms leading to vascular damage, and its elevated plasma levels were associated with endothelial dysfunction in different human diseases (71). Elevated plasma level of PTX3 have been found in patients with SSc and correlated with vascular manifestations such as digital ulcers and pulmonary hypertension (PH) (70, 72). Interestingly, EPC counts negatively correlated with circulating PTX3 levels (69). These observations suggest a potential role of PTX3 in regulating vascular homeostasis in SSc. In an experimental model, the exposure to a high concentration of PTX3 inhibited EPC differentiation. Based on these data, PTX3 seems to be an additional contributor to worsening the outcome of vascular EPC-mediated repair in SSc (70).

To further investigate these functional changes in SSc EPCs, the gene expression profiles were investigated by Avouac et al. (73). In this study, a different gene expression profile was observed in EPCs from SSc patients compared to control subjects. Interestingly, many of these genic alterations were associated with a proadhesive, proinflammatory, and activated phenotype. Furthermore, experimental hypoxia conditions modulated the gene expression profile of late-growth EPC-derived endothelial cells (73) showing a further upregulation of genes involved in inflammatory and immune response and a downregulation of HOXA9, a factor necessary for endothelial tube formation during angiogenesis (74) and a key regulator of adult progenitor cell commitment to the endothelial lineage (75).

Further studies on late-outgrowth EPC-derived endothelial cells from SSc patients have revealed that when compared with patient mature dermal microvascular endothelial cells, these EPC-derived cells may already present or not alterations in the expression of key regulators of vascular integrity and angiogenesis, such as epidermal growth factor-like domain 7 (EGFL7) (76),the key VEGF co-receptor neuropilin-1 (NRP-1), and Fli1 transcription factor (77). EGFL7 is an important pro-angiogenic molecule, almost exclusively expressed by and active on endothelial cells and their progenitors. EGFL7 expression is highest when the endothelium is in an active, proliferating state, and is a key actor in controlling vascular patterning and integrity (78). EGFL7 expression in progenitor and mature endothelial cells is deeply downregulated in SSc patients, suggesting its role in the mechanisms of defective vascular repair machinery characteristic of SSc (76). On the contrary, it has been demonstrated that SSc dermal microvascular endothelial cells exhibit impaired expression of NRP-1 due to Fli1 deficiency, while SSc late-outgrowth EPC-derived endothelial cells have a genuine phenotype characterized by normal expression levels of either Fli1 or NRP-1 (77). In another study, gene expression profiling of EPC-derived endothelial cells identified matrix metalloproteinase 10 (MMP10) as a novel candidate gene in SSc-associated PH (79). MMP10 seems to have a predominant role in pathologic conditions related to tissue repair and inflammation (80) and more recently its role in neovascularization has also been proposed (81, 82). Circulating serum proMMP10 concentrations were markedly increased in patients with SSc-associated PH compared to SSc patients without PH and healthy controls. Microarray experiments showed that the MMP10 gene was the top upregulated gene in EPC-derived ECs from patients with SSc-associated PH (79).

Another hypothesis has been made on the mechanisms underlying SSc pathogenesis. Once recruited at sites of vascular damage and after exposition to TGFβ, EPCs might transdifferentiate into myofibroblasts, which are the effector cells ultimately responsible for the severe fibrotic process in SSc. Thus, instead of promoting vasculogenic and angiogenic processes, EPCs and endothelial cells might undergo a phenotypic modification, called EndoMT (83, 84). In this context, recent studies showed the presence of cells in intermediate stages of EndoMT in vessels of lung and dermal tissues of patients with SSc (85–87).Furthermore, the treatment of human normal dermal microvascular endothelial cells with TGFβ and SSc sera induced a myofibroblast morphology and the expression of markers of myofroblasts (a-SMA and type I collagen) with downregulation of endothelial markers (CD31, VE-cadherin) (87). More recently, the occurrence of such a phenotypical change from endothelial cells to myofibroblasts has been further demonstrated in the early circulating EPCs from SSc patients (88).

## EPC-Based Therapies for SSc

In view of the fact that EPCs are defective in several chronic diseases, including SSc, different therapeutic strategies could be postulated to stimulate the production of EPCs or directly use these cells for vascular repair in these conditions. From a theoretical point of view, stem/progenitor therapies might be superior to pharmacological therapy not only because of their direct vasculogenic properties but also paracrine action related to the secretion of multiple growth factors known to be effective in promoting angiogenic processes (89).

### Pharmacological Approaches to Improve Endothelial Repair Mechanisms

Several pharmacological agents have been shown to impact on the number and function of EPCs in animal models and small clinical studies. Here, we focus on recent data concerning the effects of pharmacological agents in clinical use for the treatment of SSc vasculopathy.

3-Hydroxy-3 methylglutaryl coenzime A reductase inhibitors, or statins, have been developed as lipid lowering drugs, but besides this well-known effect, statins are capable of having anti-inflammatory and immunoregulatory effects (90). In particular, statin therapy improves endothelial function by decreasing platelet aggregation and increasing endothelial-derived nitric oxide production (91). Moreover, statins induce mobilization of EPCs from BM (92, 93), increase their functional activity and, probably, their homing to sites of vascular injury (94). The beneficial effect of statins in the treatment of different vascular diseases led several investigators to propose them as a potential treatment for SSc vasculopathy (95), although only a few studies have evaluated the clinical effect of statins in SSc patients. Two studies measured the impact of statin treatment on EPCs and mature endothelial cells (96, 97). They were both open-label studies in which all the participants received statins, and the EPC counts were made before therapy, upon completion of the trial, and throughout. Kuwana et al. treated 13 SSc patients with atorvastatin 10 mg/day for 12 weeks. The authors observed a significant improvement in peripheral vascular manifestations during the treatment period. Atorvastatin treatment resulted in a 1.7- to 8.0-fold increase in the EPC number from baseline, and the number returned to baseline after treatment with atorvastatin was stopped. Circulating levels of the angiogenic factors VEGF and basic fibroblast growth factor, which are believed to be upregulated in SSc to compensate for the inability EPCs to respond adequately to angiogenic stimuli, were significantly reduced during the atorvastatin treatment. In addition, the circulating levels of soluble vascular cell adhesion molecule-1 and E-selectin, which reflect the status of endothelial activation and injury, decreased (96).

Del Papa et al. compared the effects of simvastatin on EPC mobilization in a hypercholesterolemic group and in 20 normocholesterolemic patients suffering from the limited form of SSc. The therapy significantly increased the number of circulating EPCs in the hypercholesterolemic group, but failed to improve the EPC levels in the SSc patients, mainly in those with long-standing disease. In addition, baseline levels of mature circulating endothelial cells were significantly higher in SSc patients compared with controls and, at the end of the treatment, they were significantly decreased. Regarding other markers of endothelial activation, they found the levels of endothelial activation-related markers decreased in a statistically significant manner in the treated patients (97). The different results observed in the two studies could be partially ascribed to the different statins used and to a different selection of patients enrolled (96, 97). Anyway, the fact that EPC mobilization from BM is always reduced, in comparison with non-scleroderma controls, confirms the hypothesis that impaired vascular repair mediated by EPCs may have a role in the progression of the scleroderma vasculopathy. Different mechanisms may be postulated to explain the failure of EPC recruitment in the BM of SSc patients treated with statins. First, as observed in patients with cardiovascular risk factors, the prolonged request from the peripheral damaged vessels may induce the exhaustion of the BM reservoir of EPCs (36). Second, as other organ and tissues involved in SSc, the involvement of the BM microvascular set could interfere with the processes driving the mobilization of EPCs, including those mediated by statins.

Erythropoietin (EPO) was originally described as a hematopoietic cytokine, regulating proliferation and differentiation of erythroid precursor cells. However, several recent studies have suggested that EPO exerts other important anti-apoptotic and anti-inflammatory effects, beyond the regulation of hematopoiesis. EPO induces mobilization of EPCs from the BM as shown in animal models and humans. Heeschen et al. demonstrated that EPO treatment improved neovascularization in a murine hindlimb ischemia model, and this effect was associated with an increase in the number and proliferation of EPCs (98). These results have been confirmed in humans by the demonstration of a correlation between serum concentration of EPO and number and function of both BM-derived and circulating EPCs in patients with coronary artery disease (99). With regard to SSc vasculopathy, Ferri et al. reported an SSc patient with nonhealing cutaneous ulcers successfully treated with recombinant human erythropoietin (rHuEPO). Before rHuEPO treatment, the BM sample from this patient contained reduced numbers of EPCs, which were functionally impaired. After a 6-month rHuEPO cycle, a marked increase in endothelial progenitor markers was seen, along with a significant reduction in their apoptotic rates (100).

Furuya et al. assessed EPC counts in a small group of patients with SSc and alveolitis. They showed that low-dose i.v. cyclophosphamide (CYC) plus corticosteroid, but not corticosteroid alone, increased the EPC levels. Moreover CYC-induced EPC recruitment was less efficient in SSc patients in comparison with those with other connective tissue disease, thus confirming the well-known impaired differentiation potential of EPC in scleroderma (101).

Other EPC mobilizing cytokines are under investigation in cardiovascular diseases (i.e., G-CSF, VEGF) (94). However, their use at present is hampered by the fact that the intrinsic mechanisms, whereby they alter number and function of EPCs, should be determined in more detail.

### Transplantation of EPCs

Direct injection of EPCs into circulation or into the injury site (namely ischemic site) is a therapeutic option that has been shown effective and safe in animal models. However, although promising, little is known about the real benefit of EPC transplantation in several clinical trials. The heterogeneity of the definition and characterization of EPCs results in the use of different cell populations for vascular repair. In this context, interpretation of results from human studies cannot be definitive. In addition, most trials are pilot studies, not controlled and involving small numbers of patients. Furthermore, the rate of homing, incorporation, survival, and long-term follow-up observations are still lacking, with no results regarding the potential cancer risk and/or immunoreactivity. Finally, the consistent evidence of the impairment and reduction of EPCs in diabetes, atherosclerosis, and autoimmune diseases, represents a theoretical obstacle against cell-based therapies with autologous cells. This feature is further evident with regard to SSc, as suggested either by the *in vitro* evidences (40, 58) and the experimental demonstration of an impaired *in vivo* neovascularization capacity of EPCs from SSc patients in SCID mice (102). On the other hand, the improvement of EPC isolation and amplification techniques or the use of heterologous cord blood-derived cells might represent a valid future direction of these strategies.

With regard to SSc, similarly to other vascular diseases, the use of autologous EPCs has been largely suggested as a therapeutic option for ulcer healing and other vascular complications related to scleroderma vasculopathy. Local injections of isolated CD34^+^ cells obtained from PB after mobilization by G-CSF or isolated from BM, probably including EPCs, have been shown to be effective in inducing a rapid and evident beneficial effect on vascular symptoms and ulcer healing (103).

Adipose tissue has been also proposed as a cell source for therapeutic angiogenesis in ischemic diseases. Adipose tissue is mainly composed of two types of cells: mature adipocyte and their precursors, the so-called stromal vascular fraction (SVF) which contains multipotent mesenchymal stem cells, EPCs, pericytes, and macrophages. The ability of SVF to promote angiogenesis and neovascularization has major implications for diseases characterized by poor vascularization, ischemia, and necrosis. Its application has resulted in neovascular formation when applied to acute myocardial infarction, cosmetic procedures, burn wounds, diabetic foot ulcers, and ischemic muscle (104). Prompted by these results, the simple autologous fat grafting (AFG) and other evolved procedures have been used for the treatment of SSc cutaneous complications, such as perioral changes and hand involvement, including skin fibrosis and ulcers (105–107). The present data do not allow us to attribute the reported clinical benefit common to all the studies to a specific subpopulation of cells or a specific mechanism. However, these studies clearly provide evidence that AFG was able to induce neoangiogenesis in the lip and fingers after treatment as suggested by the significant improvement induced by lipofilling in microvascular patterns at labial and digital capillaroscopy (105, 106). Moreover, the AFG in the perioral area induced a neoproliferation of dermal capillaries and reduced the fibrotic changes with partial restoration of the dermal structure, suggesting that the local tissue improvement observed after AFG occurs *via* a pro-angiogenic process (105). To date, therapeutic mechanisms responsible for angiogenic properties of adipose tissue implantation have not been fully understood. It is likely that both the heterogeneous cellular mixture and growth factors may account for the robust angiogenic and vasculogenic potential confirmed in experimental and human studies (104).

## Conclusion

Despite the presence of several stimuli that induce the formation of new vessels such as tissue hypoxia and increased levels of VEGF, appropriate vessel repair does not occur in SSc patients. In SSc, EPCs, usually involved in the mechanisms of vascular repair, have been deeply investigated, with consistent findings showing a significant dysfunction and/or altered cell counts in both PB and BM environment.

In this scenario, a possible therapeutic strategy could be the use of drugs able to induce mobilization, homing, and proliferation of EPCs or alternatively the local or systemic use of purified EPCs or their precursors to treat microvascular damage. Pharmacological interventions with statins or growth factors have shown partially positive results. Few but encouraging studies with different tissue sources of progenitors (including EPCs, mesenchymal stem cells, pericytes) have been carried out in a limited number of patients and so their results are far from conclusive (107–110). Adipose tissue is now under investigation as an alternative source of pro-angiogenic stem cells and pilot studies have demonstrated that these cells may be useful in improving scleroderma-related fibrotic and vascular complications when grafted locally.

## Author Contributions

NNDP designed the review organization and wrote it, based on her expertise in the field. FP helped in revising the literature on the subject and writing the manuscript.

## Conflict of Interest Statement

The authors declare that the research was conducted in the absence of any commercial or financial relationships that could be construed as a potential conflict of interest.
